# Heartmate 3 left ventricular assist device system in patients with glucose-6-phosphate dehydrogenase deficiency

**DOI:** 10.1177/02676591251334897

**Published:** 2025-04-14

**Authors:** Elena Grasso, Roberto Lorusso, Ahmed Ibrahim, Mohamad Ibrahem Abdelhamed, Hassane Abdallah, Omer Ali Sayin

**Affiliations:** 1 Department of Adult Cardiac Surgery, 204603 Prince Sultan Cardiac Center- Al Hassa, Saudi Arabia; 2 Cardio-Thoracic Surgery Department, Heart & Vascular Centre, 199236 Maastricht University Medical Centre (MUMC+), Maastricht, The Netherlands; 3 Cardiovascular Research Institute Maastricht (CARIM), Maastricht, The Netherlands; 4 Department Research& Biostatistics, 204603 Prince Sultan Cardiac Center- Al Hassa, Saudi Arabia; 5 Department of Vascular Surgery, 30231 Kantonsspital, Aarau, Switzerland

**Keywords:** LVAD, G6PD deficiency, hemolysis, hemoglobinopathies

## Abstract

**Background:**

Glucose-6-phosphate dehydrogenase (G6PD) deficiency is a genetic enzymatic disorder that affects millions of people worldwide and characterized by hemolysis under oxidative stress. Left ventricular assist devices (LVADs) have substantially enhanced survival and quality of life for individuals with advanced heart failure. However, their use is associated with the risk of hemolysis, thrombosis, and embolic events. These risks may be heightened in patients with glucose-6-phosphate dehydrogenase (G6PD) deficiency. Given the limited published research on this subject, the primary objective of this study was to assess the degree of hemolysis and identify associated factors in adult patients with glucose-6-phosphate dehydrogenase (G6PD) deficiency who underwent Heartmate 3 (HM3) left ventricular assist device (LVAD) implantation.

**Methods:**

This retrospective, observational, single-center study was conducted on adult (>18 years of age) patients with G6PD deficiency, who underwent LVAD implantation using the HM3 LVAD between 2017 and 2022. Hemolysis-related investigation and findings as well as in-hospital outcome were assessed.

**Results:**

Left ventricular assist devices (LVADs) were successfully implanted in five adult patients with G6PD deficiency, including one individual with associated sickle cell trait (SCT). There were no major complications or fatalities during the hospitalization period. The average follow-up duration was 30 months (mean 30.4 ± 13). During the follow-up period, two patients died, two LVADs were explanted, and one patient received a heart transplant. No instances of macroscopic hemolysis were observed throughout the follow-up period.

**Conclusions:**

While our study was limited in size, LVADs seem safe for G6PD-deficient patients and offer significant clinical benefits. Larger studies are needed to confirm this and assess long-term interactions.

## Introduction

Durable mechanical circulatory support (MCS) and ventricular assist devices (VADs) have evolved significantly since their introduction over two decades ago. They are now considered viable options for patients with advanced heart failure who are ineligible for transplantation or unable to wait for a donor.^1,2^ However, hemolysis has become an increasingly recognized complication of LVAD_s_ support.^3^ The incidence of hemolysis varies (5%-18%) depending on the definition and among different generations of LVADs, being slightly higher in continuous-flow than to pulsatile devices.^4^ There is no reliable prediction or algorithm available for the occurrence or management of hemolysis in patients with LVADs. However, careful clinical and laboratory monitoring remain the cornerstone of patient management in this respect. G6PD deficiency is one of the common human enzyme deficiency disorders with variable prevalence in the middle east and Mediterranean countries.^5^ This condition might represent a risk factor of exacerbation of hemolysis in LVAD recipients.^6^ In order to provide a larger, albeit still very limited series, the aim of this case series was to provide a larger clinical investigation on hemolysis and related biomarkers in G6PD deficiency adult patients submitted to left VAD HeartMate 3 (HM3) at our center and followed up after hospital discharge.

## Methods

This study presents a retrospective, single-center case-control analysis of 28 HM3 LVAD implants performed at our center since 2017. Of these patients, four (14%) had G6PD deficiency and one (3%) had SCT associated G6PD deficiency. Given the high prevalence of G6PD deficiency in Saudi Arabia, routine qualitative testing was performed on all patients to confirm the presence of this enzyme deficiency. Notably, none of the patients were clinically symptomatic for G6PD deficiency.

The data presented in Table 1 were collected in all patients and retrospectively reviewed from the electronic database. The Interagency Registry for Mechanically Assisted Circulatory Support (INTERMACS) defines hemolysis as a plasma free Hgb ≥40 mg/dl in association with clinical signs of hemolysis ≥72 hours after implantation.^5,6^ For the purposes of this study, we defined hemolysis according to our institutional protocol, which includes patients with the following 3 parameters: unexplained anemia (Hgb <10 g/dl) in the absence of obvious bleeding, total bilirubin (≥1 mg /dl) and high LDH (>250 u /l). Plasma-free Hgb data was not available for most patients, as they were referred to a laboratory and were not routinely monitored during the study period. Besides, we evaluated the onset of macroscopic hematuria.

**Table 1.** Patients’ characteristics.

Variable	Patient 1	Patient 2	Patient 3	Patient 4	Patient 5	Mean ± SD
Age	55	35	26	27	26	34 ± 12
Gender	Male	Male	Male	Male	Male	NA
Blood group	O	B	O	B	B	NA
Rh	+ve	+ve	+ve	+ve	+ve	NA
BMI (kg/m^2^)	24.5	24.6	24.4	26	32	26 ± 3
Indication	DCM	DCM	DCM	DCM	DCM	NA
Diabetes mellitus	x	x	x	x	x	NA
Smoking	✓	✓	✓	x	✓	NA
INTERMACS profile	4	3	3	3	3	3.2 ± 0.4
CI (l/min/m^2^)	2.3	1.36	1.3	1.9	1.4	1.7 ± 0.4
EF (%)	20	10	10	12	12	13 ± 4
CVP (mmHg)	13	16	12	17	15	15 ± 2
MPAP (mmHg)	23	36	35	41	31	33 ± 7
PCWP (mmHg)	17	28	30	28	26	26 ± 5
TAPSE (mm)	11	12	15	11	12	12 ± 2
Preoperative ICD/Pacemaker)	x	_✓_	x	x	x	NA

Abbreviations: BMI = body mass index, DCM = Dilated cardiomyopathy, CI = cardiac index, EF = Ejection fraction, CVP = central vein pressure.

MPAP = mean pulmonary artery pressure, PCWP = pulmonary capillary wedge pressure, PCWP = pulmonary capillary wedge pressure, TAPSE = Tricuspid Annular Plane Systolic Excursion, ICD = implantable cardioverter defibrillator : ✓ = Yes , X = No, +ve: positive.

### Surgical and anesthetic management and cardiopulmonary bypass

All patients received assigned cardiac medications until the morning of surgery. G6PD positive patients were prioritized in the operating room to reduce the preoperative stress response. In the weeks leading up to the surgery, the antidiabetic therapy was modified applying an aggressive treatment against perioperative hyperglycemia (tight glycemic control). In the period of preoperative preparation of the patient, the availability of cross- matched blood products was ensured in order to be ready in the event of significant pre or perioperative hemolysis. None of the patient received blood or any blood products transfusion preoperatively. Upon arrival in the operating room, a close temperature control (intraoperative use of warming blankets) was implemented.^7^ After intubation, all patients were ventilated with 100% oxygen throughout the operation, except during the extracorporeal circulation. All invasive procedures were performed while the patients were under deep anesthesia to avoid oxidative stress.^8,9^ Blood gas checks were repetitively executed to detect acidosis and hyperglycemia, which are potential precipitating factors for hemolysis. Perioperative changes in temperature, hemodynamics, respiratory and metabolic parameters were recorded. The nasopharyngeal temperature was kept at 37°C during the entire bypass time.^9–11^ All patients underwent cardiac surgery through full median sternotomy and received the VAD system HM3. Immediately after coming off CPB, the patient urine was checked regarding any signs of hematuria. Patients were extubated when optimal cognitive, hemodynamic, and respiratory functions were achieved. For postoperative pain management, paracetamol was administered with minimal dose 20-40 mg/kg/day and low-dose aspirin (ASA) was stared on day 2 post intervention^12^ and warfarin with a target INR of 2.0-3.0 unless bleeding complications occur.

### Statistical data analysis

Data was analyzed using SPSS version, descriptive data analysis presented in form of mean ± SD, Number proportion and percentages. We assessed, biomarkers including (LDH, hemoglobin, bilirubin, Platelets) at Baseline, day1, day7, month1, month3, month6, month9, 1year1, 1.5 year, 2 years, 3 years and 4 years, statistical comparison was performed using *t*-tests. Confidence level was set at 95%, *p*-value <.05 was considered statistically significant.

## Results

Base‐line pre‐LVAD implantation patients' characteristics were presented in Table 1. Five male patients with DCM associated with G6PD were included in this study. Preoperative evaluation was conducted for each patient (Table 2). Table 3, shows intraoperative procedures. All patients underwent LVAD HM3 implantation. No blood or blood product transfusions were administered to any of the patients during the surgical procedure. Cardiopulmonary bypass induced hemolysis as observed in urine analysis assessed upon ICU arrival; only Patient two showed bypass induced hemolysis. We did not analyze the urine, which appeared bloody. Table 4 shows, post-operative parameters, the mean intraoperative time (IOT) was 39 minutes (median 22; range: 7- 48). Mean intensive care unit length of stay was about 7 days. Mean drainage was 564 mL/ 24 hours. Blood transfusion data are represented in Table 4. Anticoagulation for LVAD was started with low molecular weight heparin on the ICU after 12–24 h, depending on bleeding amount. Antiplatelet drugs and oral anticoagulants were started on the second postoperative day, unless bleeding complications occur. There was neither mortality nor complication within 30 days, 3 months and 6 months postoperatively. Fortunately, there were no in-hospital deaths.

Patient 1: surgery went very smoothly and the patient recovered and his LVAD was decommissioned after 40 months of surgery.^13^
Patient 2: There was a noticeable hematuria in the ICU. Then, the patient developed drive-line infection and his culture revealed methicillin-resistant staphylococcal (MRSA) infection. Subsequently, his condition was complicated with abdominal abscess and then cerebral hemorrhage which was the direct cause of death (died after 32 months of follow-up).
Patient 3: decommissioning was done after 12 months of surgery.^13^
Patient 4: The patient suffered right sided heart failure and advanced to hepatic failure and cerebral hemorrhage which caused his death after 16 months of surgery.
Patient 5: who had history of toxic substance intoxication and exhibited both G6PD deficiency and SCT, was the only patient in the study to undergo a heart transplant after less than two years of receiving a LVAD.

**Table 2.** Preoperative biomarkers.

Variable	Patient 1	Patient 2	Patient 3	Patient 4	Patient 5	Mean ± SD
Hb (g/dl)	13.8	16	12.6	13.6	13.2	13.8 ± 1.3
RDW	11.3	13.2	15.1	13.2	14.3	13.4 ± 1.4
White blood cell count (10^3^ μL)	5.62	10.03	6.97	12.9	9.54	9.0 ± 2.8
Platelet (10^3^ μL)	266	248	621	236	225	319 ± 169
INR(s)	0.88	1.21	1.38	1.09	1.01	1.11 ± 0.2
Urea mmol/L *(normal range 2*.*5-6*.*4)*	8.7	5.99	7.26	9.20	5.67	7.36 ± 1.6
Creatinine *μmol/l (normal range 62-115)*	120	75	72	76	95	87.6 ± 20.3
Uric acid *μmol/l (normal range 155-357)*	491	197	292	490	141	322.2 ± 162.8
LDH U/L (normal range 135-225)	142	224	165	201	220	190 ± 42.5

Abbreviations: RDW (Red blood cells Distribution Width) male adult normal range 11.8-15.6%, female adult normal range 11.9-15.5% , LDH = lactate dehydrogenase.

**Table 3.** Intraoperative procedures.

Variable	Patient 1	Patient 2	Patient 3	Patient 4	Patient 5	Mean ± SD
Implanted device	LVAD HEART MATE III	LVAD HEART MATE III	LVAD HEART MATE III	LVAD HEART MATE III	LVAD HEART MATE III	NA
CPB min	125	108	83	108	85	102 ± 18
Cross clamp min	0	0	0	0	0	0
Cardioplegia	0	0	0	0	0	0
Temperature (^o^C)	37	37.2	37	37	37.3	37.1 ± 0.1
Blood transfusion						
PRBC	0	0	0	0	0	0
FFP	4	2	2	2	4	3 ± 1.1
PTL	3	2	1	2	2	2 ± 0.7
Urine hemolysis after CPB	0	1	0	0	0	0.2 ± 0.4

Abbreviations: CPB = cardiopulmonary bypass, PRCB = Packed red blood cells: FFP = Fresh frozen plasma, PTL = platelet.

Abbreviations: IOT = intubation intraoperative time, ICU = intensive care unit, ✓ = Yes, X = No.

**Table 4.** Post-operative parameters.

Variable	Patient 1	Patient 2	Patient 3	Patient 4	Patient 5	Mean ± SD
OTI time h	7	24	24	8	48	22 ± 17
ICU stay in days	5	8	6	9	5	7 ± 1.8
Hypoxia	x	x	x	x	x	NA
Draining ml/ 24H	550	750	540	520	460	564 ± 110
Transfusion 24h	4	4	2	3	2	3 ± 1
PRBC	2	2	3	2	3	2.4 ± 0.5
FFP	1	0	1	1	0	0.6 ± 0.01
Urine hemolysis	x	x	x	x	x	NA
Length of hospital stay after LVAD	17	23	13	22	34	21.8 ± 8
Follow-up duration (months)	48	32	35	16	21	30.4 ± 13
Survival status	✓	x	✓	x	✓	NA

Finally, patients 1 and 3 who had lower preoperative LDH, lower bilirubin levels, and higher follow-up Hb had improvement in heart failure leading to VAD deactivation, at 40 months and 12 months, respectively months after implantation.

## Discussion

This case series of five patients with G6PD deficiency and chronic heart failure receiving durable VADs represents the first reported experience with a suggested management pathway for this combination in patients undergoing LVAD implantation. It is estimated that about 400 million people are affected by G6PD deficiency worldwide.^14^ This genetic condition is inherited as an X-linked recessive state, and presents one of the most frequently encountered red-cell enzymopathies. The major interest in the G6PD-deficient state results from the associated hemolytic anemia resulting from oxidative stress.^15,16^ It is worth mentioning that, hemolysis-induced thrombotic events in G6PD patients occur when hemolysis releases free hemoglobin into the bloodstream. This free hemoglobin binds to haptoglobin, forming methemoglobin. When haptoglobin is saturated, free methemoglobin can precipitate in the microcirculation. These precipitates can activate platelets and clotting factors, leading to thrombus formation.^14^ As mentioned, mechanical circulatory devices have been shown to induce, in some occasion, marked shear stress and, subsequently, blood elements damage.^15–17^

In our center we have implanted only HM3 devices since 2017.

The HM3 is a third-generation continuous flow centrifugal LVAD with the rotor suspended in the bloodstream using a non-contact design via magnetic levitation. The main advantages of this type of system are non-contact bearings, easy changes in pump speed to modulate an artificial pulse, and the smaller size of the device

When it comes to the effect of the LVAD device itself, the HeartMate 3 LVAD is renowned for its superior hemocompatibility, which is attributed to its innovative design features. These include a magnetically levitated rotor that minimizes blood damage, textured blood-contacting surfaces that reduce shear stress, and optimized fluid dynamics that promote smooth blood flow. These design elements contribute to a reduced risk of hemolysis, thrombosis, and other blood-related complications, ultimately improving patient outcomes.^7,18,20^

**Figure 1.**
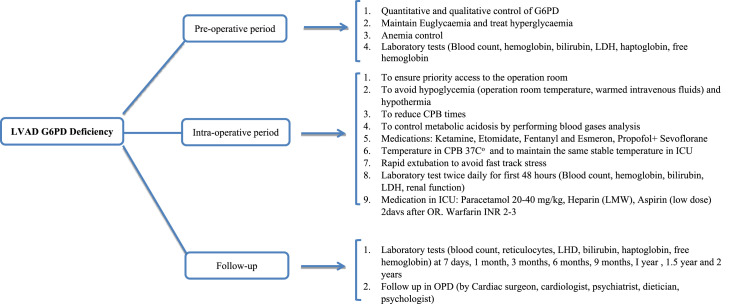
Follow chart of LVD implantation in patient with G6P Deficiency.

In fact, according to Uriel N.et al. in the secondary analysis of the MOMENTUM 3 study, HM3 Left ventricular assist System (LVAS) demonstrated greater freedom from HRAEs (hemocompatibility-related clinical adverse events) compared to HMII LVAS at 6 months.^21^ We used this LVAD in all patients in the series and ventured to include it also in a restricted population with G6PD deficiency. From the analysis in our possession, we have highlighted that for the best management of this small number of patients it would be useful to propose a management flow chart (Figure 1). From our limited case series, patients with G6PD who are candidates for LVAD implantation must follow a dedicated pathway as those with sickle cell disease should.^22^ In particular, initially all patients must absolutely avoid postoperative hypoxemia (Spo2 ≤ 90% or a Po2 ≤ 60 mmHg). VAD implantation requires the use of extracorporeal circulation which activates the inflammatory response.^18,23^ One of the end results of this process is endothelial damage. Apparently, the lungs are the main organ affected and sustained by this damage.^10^ A study by Gerrah et al.^11^ indicates that CPB is a causal factor of hypoxia in patients with G6PD deficiency, leading to increased hemolysis. As a result, these patients lose lung oxygenation reserve, as seen with a lower than normal minimum PaO2 individuals. To avoid this, we shortened CPB times and extubated patients relatively quickly to avoid the increase in free radicals that would be added to those undergoing long-term CPB.^24^

Secondly, anemia (HB less than 8 g/dl) should be avoided in the pre-, intra- and postoperative periods. Our patients did not receive red blood cell transfusions in the pre- and intraoperative period, and we used the cell salvage system with the aim of avoiding or limiting any predisposing factors related to hemolysis. Furthermore, hypothermia, commonly established during cardiac surgery, also constitutes a risk factor for hemolysis in patients with G6PD deficiency. Gerrah et al.,^11^ in their study of 42 patients, described a positive correlation between hypothermia and postoperative hemolysis. The average temperature during bypass in the study group was 26.7 ± 2.4°C. In our patients, the nasopharyngeal temperature was 32-34°C during the bypass period, and our patients did not show any signs of hemolysis. The link between G6PD deficient and hemolysis in the context of mechanical circulatory support (MCS) is not entirely new^19^ and we hypothesize that hemolysis resulted, at least in part, from an unfavorable oxidative environment after LVAD implantation rather than from shear stress alone.^23^ Indeed, it may be useful in the future to obtain peripheral blood smears (Heinz bodies and bite cells) which in many of these patients would confirm oxidative hemolysis rather than mechanical destruction, an analysis that should be performed pre- and post-operatively (Figure 1). Hemolysis is an important complication of continuous flow LVADs in all patients.^4^ Ravichandran et al.^6^ demonstrated that hemolysis after LVAD is associated with a high 1-year mortality that is more than 2 times greater than that observed for the non- hemolyzing HM3 patients. In addition, in light of our current findings, we now use aspirin 81 mg and warfarin with a goal INR of 2.0–3.0 unless bleeding complications arise. High doses of ASA were not allowed in deficient patients but low dose of ASA cause no hemolysis.^12^ We also dose bridging anti-coagulation until an INR of two is achieved.^20,25^

Furthermore, our results suggest that G6PD deficiency does not negatively impact post-implant outcomes or increase the risk of hemolysis-related complications in LVAD patients. No significant increase in hemolysis biomarkers was observed during in-hospital follow-up or post-discharge, and no major hemolysis-related complications occurred in these patients.

It was observed that, Patient 4 with higher mean bilirubin, lower mean Hb and lower mean PTL associated with the smoking factor, was the only one who died from a thromboembolic event. Interestingly, we didn’t find any correlation with smoking but aggressive smoking cessation should be used in all patients with LVAD support.^3^

Finally, based on our experience, we believe that the following concepts are beneficial in the management of patients with G6PD deficiency and undergoing LVAD implantation, despite the small sample size a single-center design:

1. Establish a pre- and post-operative management protocol for the category of patients with G6PD deficient.
2. Anyone suspected of G6PD deficiency, with a family history of the disorder, a history of hemolysis, and/or of African, southern European, Middle Eastern, southeast Asian, or central and southern Pacific Island descent, should be screened for G6PD deficiency.
3. Avoid perioperative hypothermia, acidosis, hyperglycemia and postoperative infection can precipitate haemolysis in the G6PD-deficient patient.
4. LDH, bilirubin, and haptoglobin, are strong markers for hemolysis and can be regularly obtained to survey for this potentially deadly diagnosis.

These findings may have implications for the evaluation of LVAD candidacy and may suggest more intensive postoperative surveillance for patients implanted with G6PD deficiency as we described in proposed the flow chart.

## Conclusion

In addition to oxidative stress, our study also considered the effects of mechanical stress exerted by the LVAD on patients with G6PD deficiency and chronic heart failure. We demonstrated that LVADs can be safely used in this patient population by implementing a more controlled approach and stringent support protocols to minimize complications. (Table 5 and 6).

**Table 5.** Short- term follow-up for LDH level (U/L).

LDH level (U/L)	Pt 1	Pt 2	Pt 3	Pt 4	Pt 5	Mean	SD	*p* Value	Interpretation
Preop	142	224	165	201	220	190	36	0.0001	Statistically significant increase in level of LDH
day 1	405	414	300	383	570	414	98		
day 7	286	342	231	220	416	299	82	0.14	Decrease not statistically significant
1 month	202	237	204	289	237	234	35		
3° month	210	257	170	257	186	216	40	0.46	Decrease not statistically significant
6° month	198	262	173	218	171	204	38		
9° month	189	210	158	204	144	181	29	0.32	Increase not statistically significant
1° year	198	219	138	253	216	205	42		
1.5° year	176	520	192	262	270	284	138	0.47	Decrease not statistically significant
2° year	154	288	192		136	232	68		
3° year	148		121		270	218	79	0.55	Increase not statistically significant
4° year	208		186		270	243	44

**Table 6.** Short- term follow-up for total bilirubin level (μmol/l).

Total bilirubin	Patient 1	Patient 2	Patient3	Patient 4	Patient 5	Mean	SD	*p* Value	Comment
Preoperative	6.5	26.1	8.8	27	26.5	19.0	10	0.0007	Statistically significant increase
Postoperative day 1	45.9	60.6	32.7	95.6	58.8	58.7	23		
Postoperative day 7	14.9	59	10.9	31.5	73.4	37.9	27	0.12	Not statistically significant decrease
Postoperative 1 month	12.3	16.6	10.2	25.4	18.8	16.7	6		
Postoperative 3 month	5.1	13.6	6.8	37.3	16	15.8	13	0.7	Not statistically significant decrease
Postoperative 6 month	7	10.2	6.7	34	13.2	14.2	11		
Postoperative 9 month	10.6	10.2	9.9	45.7	38.5	23.0	18	0.5	Not statistically significant decrease
Postoperative I year	9.6	12.9	7.3	42.3	14	17.2	14		
Postoperative 1.5 year	8.2	55.4	11.7	147.4	32.4	51.0	57	0.2	Not statistically significant decrease
Postoperative 2 year	8.6	11.7	11.7	^a^	18.1	12.5	4		
Postoperative 3 year	7.5	27.5	7.8	^a^	31	18.5	13	0.9	Not statistically significant decrease
Postoperative 4 year	6.6	27.5	11.3	^a^	30.9	19.1	12		

^a^The values of bilirubin for Patient 4 were not included because the patient (died) or lost from follow-up .
